# SARS-CoV-2 meta-interactome suggests disease-specific, autoimmune pathophysiologies and therapeutic targets

**DOI:** 10.12688/f1000research.25593.1

**Published:** 2020-08-17

**Authors:** Gianmarco Bellucci, Chiara Ballerini, Rosella Mechelli, Rachele Bigi, Virginia Rinaldi, Roberta Reniè, Maria Chiara Buscarinu, Sergio E. Baranzini, Lohith Madireddy, Giuseppe Matarese, Marco Salvetti, Giovanni Ristori

**Affiliations:** 1 Department of Neurosciences, Mental Health and Sensory Organs, Sapienza University of Rome, Rome, 00189, Italy; 2 San Raffaele Roma Open University; IRCCS San Raffaele Pisana, Rome, 00166, Italy; 3 Department of Neurology, Weill Institute for Neurosciences, University of California San Francisco, San Francisco, California, 94158, USA; 4 Dipartimento di Medicina Molecolare e Biotecnologie Mediche, University of Naples Federico II, Naples, 80131, Italy; 5 Istituto di Endocrinologia e Oncologia Sperimentale, Consiglio Nazionale Delle Ricerche (IEOS-CNR), Naples, 80131, Italy; 6 Neuromed: IRCCS Istituto Neurologico Mediterraneo (INM), Pozzilli, 86077, Italy

**Keywords:** SARS-CoV-2, COVID-19, protein-protein interaction, autoimmune disease, interferon, virus, repurposing

## Abstract

**Background: **Severe coronavirus disease 2019 (COVID-19) is associated with multiple comorbidities and is characterized by an auto-aggressive inflammatory state leading to massive collateral damage. To identify preventive and therapeutic strategies against severe acute respiratory syndrome coronavirus 2 (SARS-CoV-2), it is important to ascertain the molecular interactions between virus and host, and how they translate into disease pathophysiology.

**Methods: **We matched virus-human protein interactions of human coronaviruses and other respiratory viruses with lists of genes associated with autoimmune diseases and comorbidities associated to worse COVID-19 course. We then selected the genes included in the statistically significant intersection between SARS-CoV-2 network and disease associated gene sets, identifying a meta-interactome. We analyzed the meta-interactome genes expression in samples derived from lungs of infected humans, and their regulation by IFN-β. Finally, we performed a drug repurposing screening to target the network’s most critical nodes.

**Results: **We found a significant enrichment of SARS-CoV-2 interactors in immunological pathways and a strong association with autoimmunity and three prognostically relevant conditions (type 2 diabetes, coronary artery diseases, asthma), that present more independent physiopathological subnetworks. We observed a reduced expression of meta-interactome genes in human lungs after SARS-CoV-2 infection, and a regulatory potential of type I interferons. We also underscored multiple repurposable drugs to tailor the therapeutic strategies.

**Conclusions: **Our data underscored a plausible genetic background that may contribute to the distinct observed pathophysiologies of severe COVID-19. Also, these results may help identify the most promising therapeutic targets and treatments for this condition.

## Introduction

The severe acute respiratory syndrome coronavirus 2 (SARS-CoV-2) is responsible for the current coronavirus disease 2019 (COVID-19) pandemic. The number of COVID-19 confirmed cases worldwide exceeds 14.5 million as of July 20, 2020, while the estimated global case fatality rate stands at 4.3%. Since the early phases of the pandemic, comorbidities such as hypertension, diabetes, cardiovascular disease, and obesity were readily identified as conditions associated with the severity of COVID-19 and the most aggressive collateral damage ^1, 2^. The acute respiratory distress syndrome was recognized as the condition that dominated the clinical picture in critically ill patients ^3^.

SARS-CoV-2 is the longest known ssRNA virus ^4^. Like its predecessors SARS-CoV and MERS-CoV that caused epidemics in 2003 and 2012, SARS-CoV-2 is highly virulent. They all belong to the Coronaviridae family, together with four other HCoVs (HCoV-229E, HCoV-NL63, HCoV-OC43, HCoV-HKU1) that, in most cases, cause common colds. SARS-CoV-2 displays an overall 79% genome similarity with SARS-CoV, reaching a 99.7% homology within the Spike protein (S) gene, the major determinant of viral tropism ^5^. In both viruses, the Spike protein binds the membrane protein angiotensin-converting-enzyme 2 (ACE2) and engages the serine protease TMPRSS2 to infect human cells ^6^. The 0.3% genomic divergence in the critical (S)-Receptor Binding Domain ^7^, and the potential to employ other proteases (e.g. FURIN) ^8^ seem to explain the strikingly higher transmission capability of SARS-CoV-2.

To identify preventive and therapeutic strategies against COVID-19, it is important to ascertain the interactions between virus and host, and how they develop into disease pathophysiology. The definition of physical interactions between SARS-CoV-2 proteins and host proteins has advanced considerably in recent weeks ^9, 10^. In order to select relevant therapeutic targets and processes, it would be meaningful to pinpoint those interactions that are key to the development of the (most severe) clinical manifestations of the disease and understand their pharmacological relevance. Furthermore, if a plausible genetic association is identified, this significantly increases the probability of success in drug development ^11^.

A series of the clinical manifestations of COVID-19 appear to be related to an auto-aggressive immune dysregulation ^12^. This evidence was clear enough to encourage the repurposing of drugs approved for the therapy of autoimmune conditions, for the treatment of severe COVID-19. It is therefore reasonable to hypothesize that the SARS-CoV-2-host interactome contains the genetic “seeds” and the therapeutic targets of the auto-aggressive processes that characterize COVID-19 (in particular the severe forms of the disease). To investigate both, we matched the SARS-CoV-2 interactome with lists of genes that genome-wide association studies (GWAS) defined as associated with major autoimmune diseases. We used this approach to also identify genes and processes that may influence the course of COVID-19 through the presence of coexisting diseases (i.e. matching the SARS-CoV-2 interactome genes with genes associated with COVID-19-relevant comorbidities).

To increase the interpretability and the specificity of our results, we also performed the above analyses using interactomes of the other coronaviruses (HCoVs) and other viruses causing respiratory diseases in humans.

## Methods

### Virus- Host interaction protein networks

We considered the interactomes from 7 human infecting members of the Coronaviridae family and from other viruses causing respiratory diseases (Influenza A H1N1, Influenza A H7N9, Influenza B, HRSV) ( *Extended data* ^13^).

The SARS-CoV-2 host-protein network was recently uncovered by an affinity-purification mass spectrometry experiment ^9^. Other host-pathogen protein interaction maps were derived from p-HIPSTER, a structure-informed atlas of human-virus interactions ^14^, which contains both interactions present in the RCSB Protein Data Bank and predicted pathogen-host PPIs.

### SARS-CoV-2 network expansion

We performed the expansion using the latest upgrade of Human Integrated Protein-Protein Interaction rEference (HIPPIE) ^15^ which integrates confidence scored and functionally annotated human protein-protein interactions (PPIs) from the public databases BioGRID, DIP, MIPS, HPRD, IntAct, MINT and BIND.

To guarantee the interaction confidence, we accounted only for physical interactions, starting from a very high PPI confidence score (0.95), and progressively expanded the network coverage until reaching a medium confidence score of 0.65 (second quartile of the HIPPIE score distribution).

### Disease-associated genes and statistical enrichement analysis

We extracted, from the NHGRI-EBI Catalog of human genome-wide association studies ^16^, the mapped genes of SNPs significantly associated (p-value ì≤ 5 × 10 ^−8^) with immune-mediated disorders [multiple sclerosis (MS), systemic lupus erythematosus (SLE), rheumatoid arthritis (RA), Crohn’s disease (CD), ulcerative colitis (UC), type 1 diabetes mellitus (T1D)] and with conditions being relevant to COVID-19 clinical manifestations and prognosis [obesity, aging, type 2 diabetes mellitus (T2D), asthma, chronic obstructive pulmonary disease (COPD), hypertension and coronary artery disease (CAD)]. Specifically, we searched for the corresponding trait in the database and collected the reported loci ( *Extended data* ^13^).

We overlapped the interactomes and the disease-associated genes and evaluated the statistical significance using *geneOverlap* package version 1.24.0 ^17^ in R, version 3.6.3. We took into consideration the Jaccard index, which represents the similarity between two sets; p-value significance cutoff was set at p<0.05 after correction with Benjamini-Hochberg method.

### COVID-19 meta-interactome construction and pathway analysis

We selected the genes included in the statistically significant intersection of SARS-CoV-2 network and disease associated gene sets, constructing a COVID-19 meta-interactome. To better understand the immune mechanism shared by COVID-19 and immunological diseases, we also defined a COVID-19 immunological subnetwork collecting genes from immune-mediated disease only.

We then performed a functional enrichment analysis of each significant overlapping subset of genes among GO Biological Processes and Reactome Gene sets using Metascape ^18^, a web-based utility. This tool uses the MCODE (Molecular Complex Detection) algorithm to perform module analysis and detect dense regions of protein interaction networks ^19^. Network visualization and node connection analysis was performed with Cytoscape 3.8.0 ^20^.

The Circle plot representing COVID-19 meta-interactome genes was produced using the Circos table viewer tool ^21^. The UpSet plot was produced using the UpSetR package for R ^22^, version 1.4.0.

### Gene expression analysis

We analyzed the COVID-19 meta-interactome genes using the GENE2FUNC of the FUMA tool ^23^, an integrative web-platform to perform post-GWAS annotation. We specifically performed a gene expression analysis based on GTEx v8 and Ensemble version v92, retrieving tissue specificity. Gene-enrichment statistical analysis was set as follows: the adjusted P value cut-off was 0.05, minimum overlapping genes with gene-sets was 2, method for multiple test correction was Benjamini-Hochberg (FDR).

We looked for the expression of COVID-19 meta-interactome genes in samples derived from lungs of infected humans. To do this, we selected from the NCBI Gene Expression Omnibus Database ^24^ the series GSE147507 ^25^, including transcriptional profiling of post-mortem lung samples of two male COVID-19 patients compared with healthy lung biopsies, and transcriptional profiling of normal human bronchial epithelial (NHBE) cells after the treatment with human IFN-I-Β. 

Briefly, at first we extracted the complete results using the GEO RNA-seq Experiments Interactive Navigator (GREIN) web tool ^26^; we then selected the COVID-19 meta-interactome genes and plot the final data with EnhancedVolcano R package, version 1.4.0.

To assess the potential of COVID-19 meta-interactome genes of being regulated by Type 1 interferon-β, we exploited Interferome 2.0.1, an online database of interferon-regulated genes (IRGs) ^27^. The search settings were as follows: Interferon type I, subtype IFN-β, any treatment concentration and time, *in vitro* and *in vivo* cell lines derived from *Homo sapiens*, any sample type, Fold change cutoffs 1.0.

### COVID-19 meta-interactome drug target screening

To assess the therapeutic potential of COVID-19 meta interactome, we used the Scalable Precision Medicine Oriented Knowledge Engine (SPOKE) ^28^. SPOKE is a heterogeneous knowledge network that includes data from 29 publicly available biomedical databases. We searched the SPOKE Neighborhood Explorer for the COVID-19 meta-interactome genes with the highest Betweenness Centrality score in the whole network and in subnetworks, filtering results with the following settings: “compound” and “gene” nodes; “compound-binds-protein”, “compound-downregulates-gene”, “compound-upregulates-gene”. We filtered nodes representing compounds at development phase ≥3; Edges Attributes and Limits were as set from the database. 

## Results

### SARS-CoV-2 interactors enrichment

In this work, we downloaded the host-protein networks of SARS-CoV-2, six other HCovS (SARS-CoV, MERS-CoV, HCoV-229E, HCoV-NL63, HCoV-OC43, HCoV-HKU1) and four other viruses causing respiratory diseases in humans (Influenza A H1N1, Influenza A H7N9, Inluenza B, Human Respiratory Syncitial Virus). We extended the experimentally determined SARS-CoV-2-host interactions ^9^ by including second neighbors, i.e. molecules directly interacting with the host proteins engaged by the virus extracted from HIPPIE. We progressively lowered the confidence score threshold to construct 7 more sets of increasing size ( *Extended data* ^13^).

We then looked for an enrichment of human proteins interacting with SARS-CoV-2 within sets of genes mapped in genome-wide studies of immune-mediated conditions and comorbidities impacting on COVID-19 prognosis.

**Figure 1.**
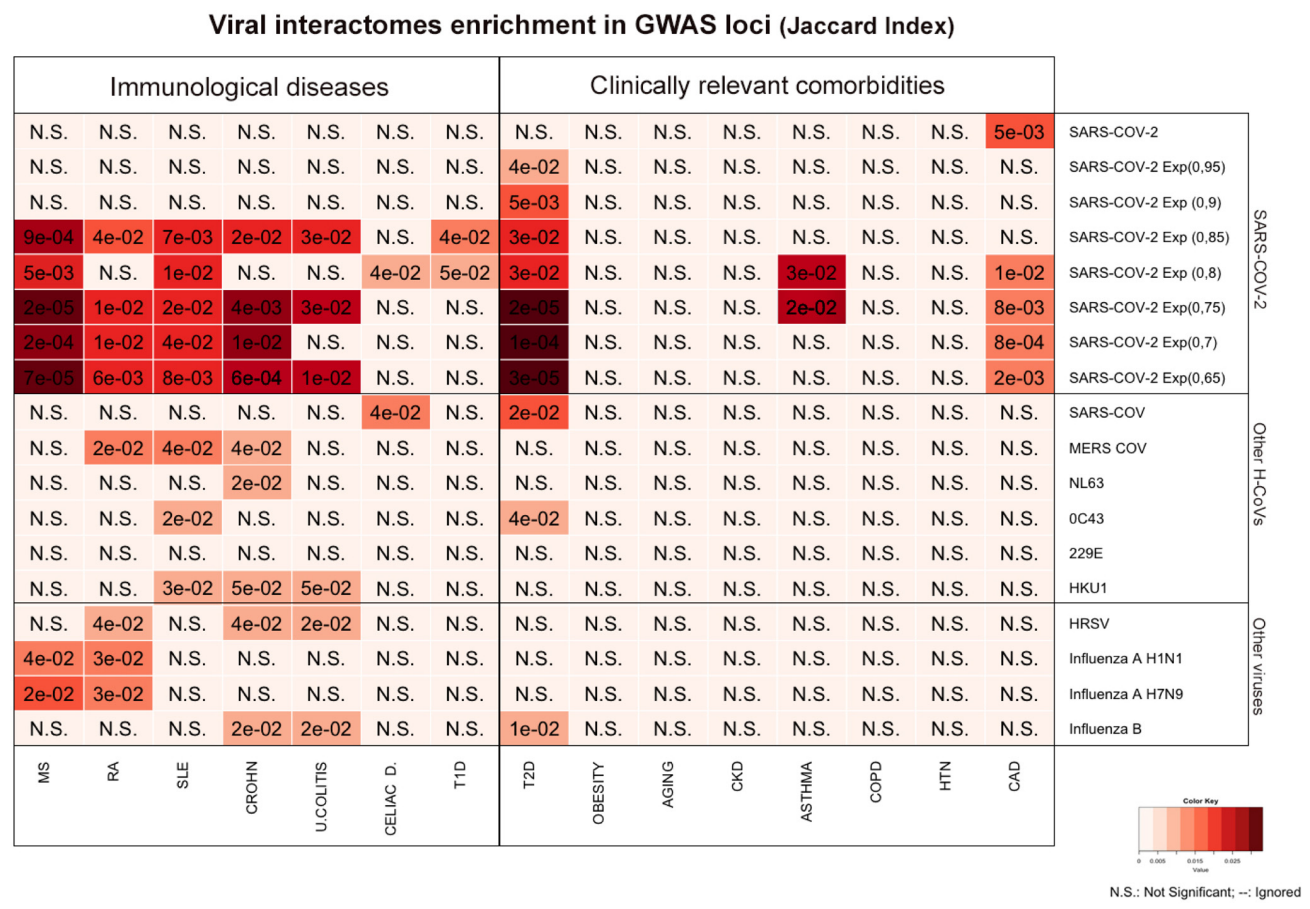
Viral interactomes enrichment in GWAS loci. The heatmap shows the Jaccard Index of the overlaps of gene sets associated with immunological diseases and COVID-19 relevant comorbidities with viral interactomes. SARS-CoV-2 section shows in the first line the host protein network derived from experimental evidence ^9^, followed by results obtained overlapping the expanded networks generated with HIPPIE settings at different confidence levels (0,95 to 0,65). HRSV: Human Respiratory Syncitial Virus; MS: Multiple Sclerosis; RA: Rheumatoid Arthritis; SLE: Systemic Lupus Erythematosus; CROHN: Crohn’s Disease. U.COLITIS: Ulcerative Colitis; CELIAC D.: Celiac Disease; T1D: Type 1 Diabetes Mellitus; T2D: Type 2 Diabetes Mellitus; CKD: Chronic Kidney Disease; COPD: Chronic Obstructive Pulmonary Disease; HTN: Hypertension; CAD: Coronary Artery Disease.

The analysis revealed a significant enrichement of SARS-CoV-2 interactome with genes associated with several autoimmune diseases ( Figure 1). The strongest significance was reached by SARS-CoV-2/MS overlap (p=0.0002), consolidated at multiple expansion levels; significant associations with CD, SLE and RA were of weaker magnitude.

When looking at clinically relevant comorbidities, there was a significant overlap between SARS-CoV-2 interactors and T2D associated genes, that followed a positive trend in parallel to the network expansion, with a maximum of 65 shared interactors ( Figure 1 and *Extended data* ^13^). The other COVID-19 relevant comorbidities reaching statistical significance were CAD and Asthma.

Neither the other HCovs nor the other respiratory viruses (H1N1, H7N9, Influenza B, HRSV) reached significance levels as high as SARS-CoV-2.

In summary, SARS-CoV-2 host protein network is enriched in sets of genes associated with multiple autoimmune diseases and shares significant interactions with three conditions influencing COVID-19 prognosis: asthma, T2D and CAD.

### COVID-19 meta-interactome construction and analysis

**Figure 2.**
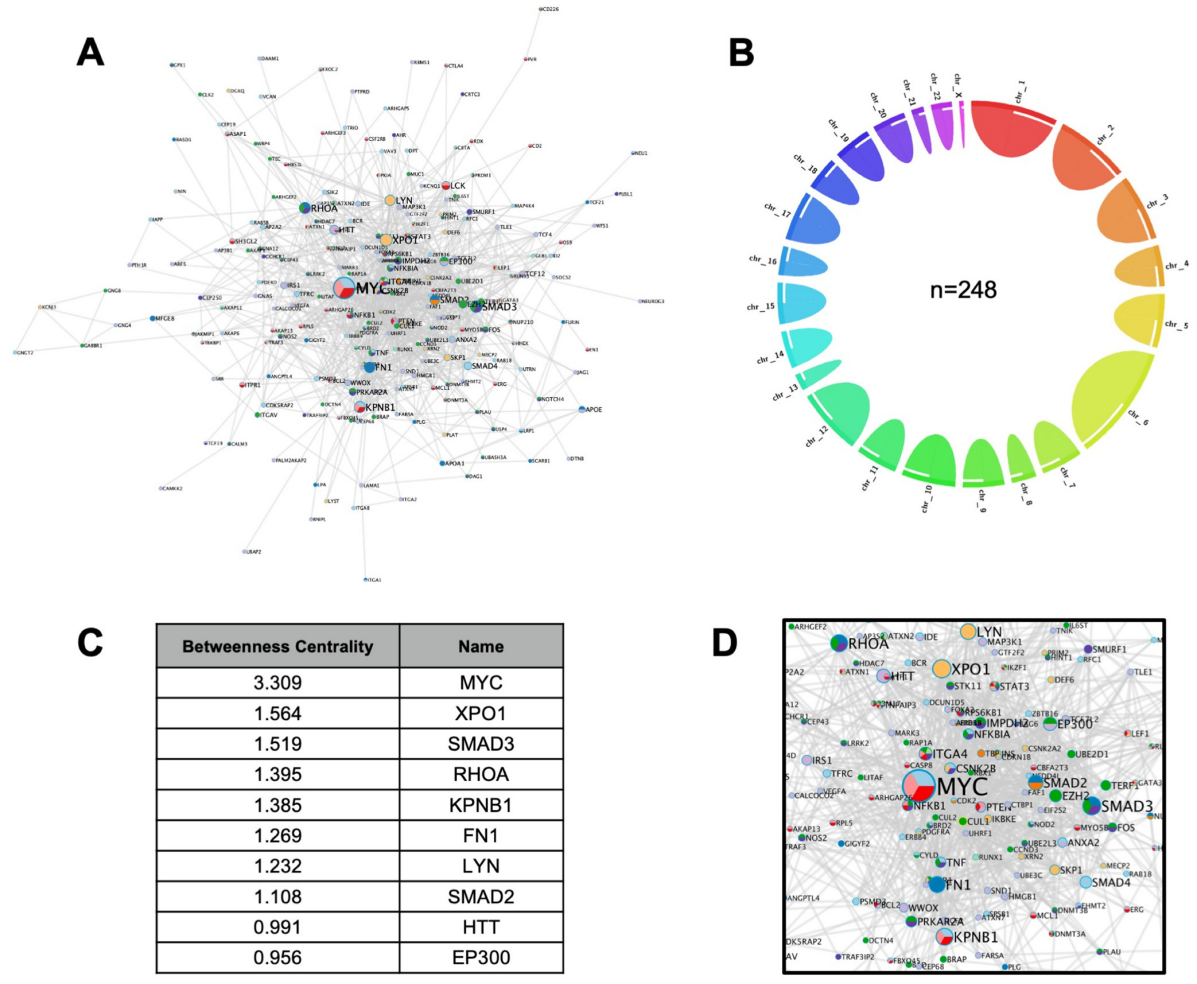
COVID-19 meta-interactome. **A**) Network Representation: node size is proportional to Betweenness Centrality score. **B**) Circle plot showing the chromosome distribution of the 248 COVID-19 meta-interactome genes. **C**) Top 10 nodes ranked by Betweenness Centrality **D**) Detail of the network view showing the top nodes.

We next constructed a COVID-19-human meta-interactome, selecting genes deriving from the significant overlaps with immunological diseases and comorbidities. The resultant network was composed of 248 genes ( Figure 2A and Extended data ^13^), with a non-specific distribution on autosomes, and a single gene located on Chromosome X ( *MECP2)* and none in chromosome Y ( Figure 2B).

**Figure 3.**
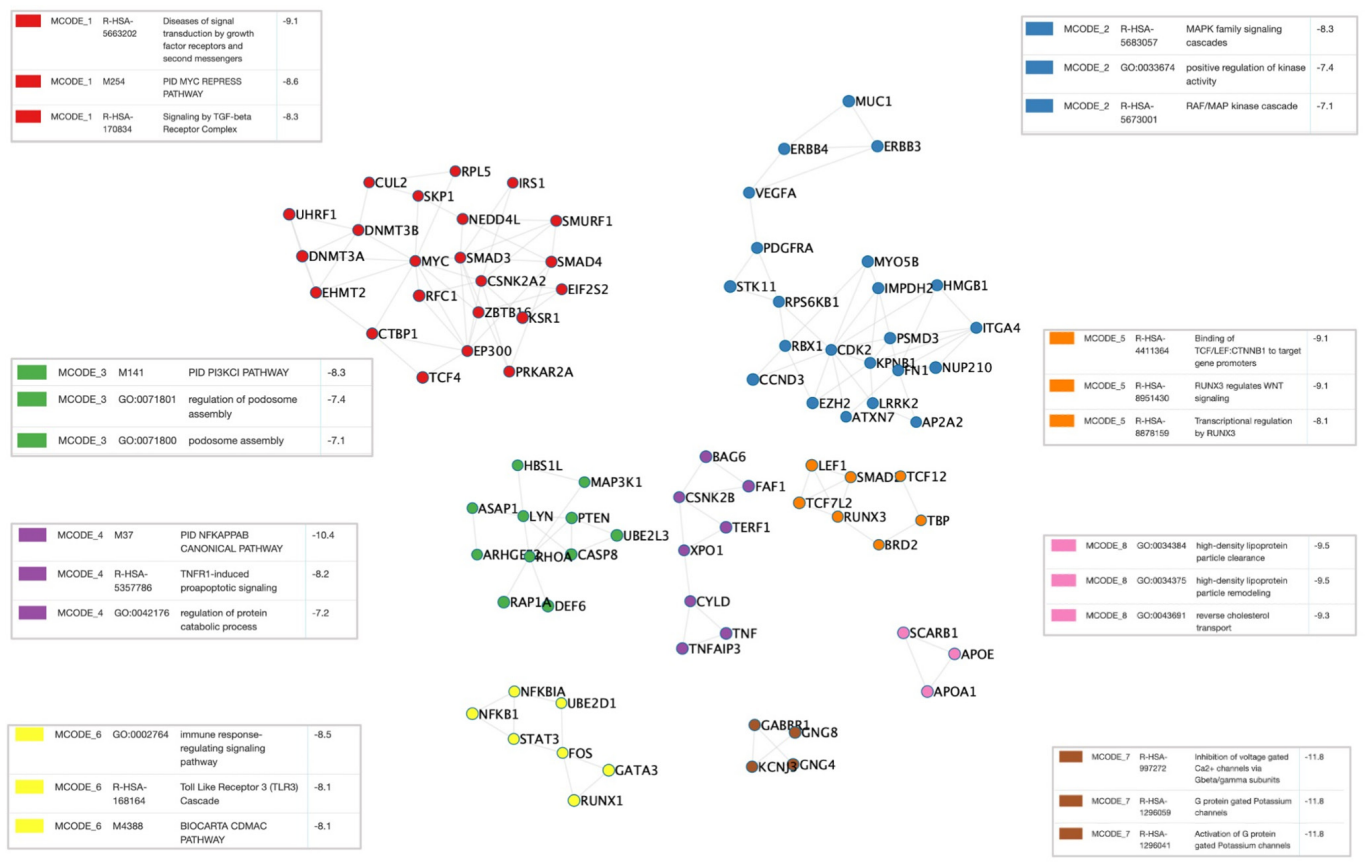
COVID-19 meta-interactome clusters. The M-CODE algorithm was applied to detect densely interconnected node subsets. The network representation displays a different color for each cluster, associated with the enriched biologic processes in the relative boxes.

**Figure 4.**
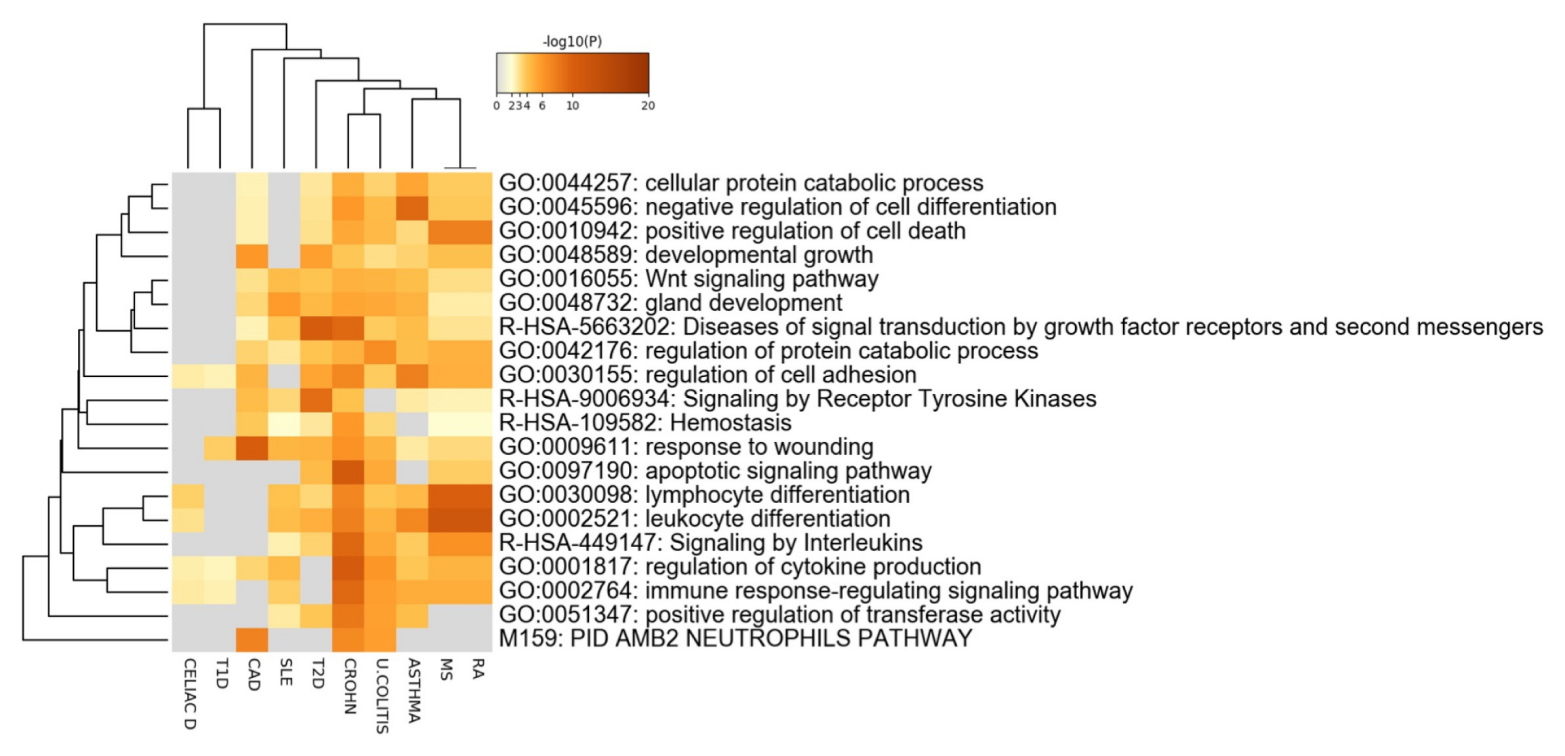
COVID-19 meta-interactome pathway analysis. Heatmap of top 20 enriched terms across input gene lists, colored by p-values. MS: Multiple Sclerosis; RA: Rheumatoid Arthritis; SLE: Systemic Lupus Erythematosus; CROHN: Crohn’s Disease. U. COLITIS: Ulcerative Colitis; CELIAC D.: Celiac Disease; T1D: Type 1 Diabetes Mellitus; T2D: Type 2 Diabetes Mellitus; CAD: Coronary Artery Disease.

To assess the prominence of interactors within the network, we ranked the gene according to the Betweenness score, a network centrality metric which reflects the number of shortest paths that go through a given node, thus representing the importance of that node on the information flow through the network ( Figure 2C). Top ranking nodes were: *MYC*, a widely acting oncogene with a key role in immune regulation; *XPO1*, whose encoded protein mediates the nuclear export of cellular proteins and of RNAs, including viral ones; *SMAD3*, an intracellular transducer of transforming growth factor-β signaling pathway ( Figure 2D). We then performed a functional enrichment analysis of each significant overlapping subset of genes using Metascape ^18^ a web-based utility which uses the MCODE (Molecular Complex Detection) algorithm, to perform module analysis and detect dense regions of protein interaction networks ^19^. Densely interconnected genes subsets pertain to central immunological processes (NFĸB, immune response and TLR3 pathway, podosome assembly, MAPK cascade), cell cycle and transcriptional regulation (TCF/LEF and WNT pathway), cholesterol and metabolism, and ionic channels regulation ( Figure 3). In line with this data, the COVID-19 meta-interactome pathway enrichment analysis ( Figure 4) showed that the top 20 clusters belonged to immunological (lymphocyte differentiation, neutrophils pathway, immune response-regulating signaling pathway, regulation of cytokine production), and cell cycle processes; interestingly, we found an enrichment of the hemostasis process. This is in line with evidence showing a coagulation dysfunction in severe COVID-19 ^29^

**Figure 5.**
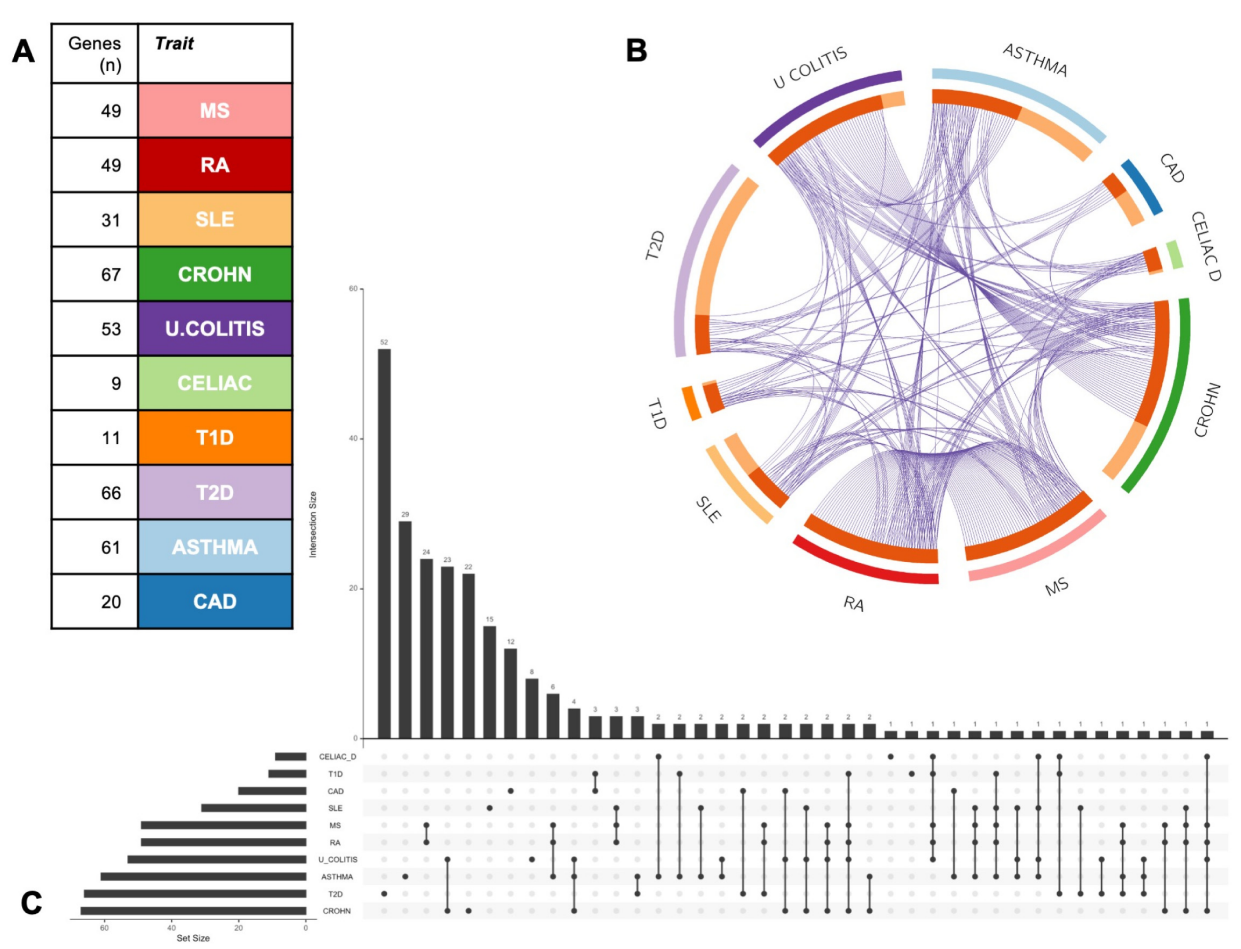
COVID-19 meta-interactome composition. **A**) The table shows the subsets of genes from each trait contributing to the network composition. **B**) The circle plot shows the overlap between gene lists at the gene level, with purple curves linking identical genes. **C**) The UpsetPlot displays a schematic representation of the intersections among gene sets (x axis), with the respective size (y axis). MS: Multiple Sclerosis; RA: Rheumatoid Arthritis; SLE: Systemic Lupus Erythematosus; CROHN: Crohn’s Disease. U. COLITIS: Ulcerative Colitis; CELIAC D: Celiac Disease; T1D: Type 1 Diabetes Mellitus; T2D: Type 2 Diabetes Mellitus; CAD: Coronary Artery Disease.

Notably, in COVID-19 meta-interactome composition, most of the genes deriving from autoimmune diseases’ sets appeared to be shared among the traits: the most relevant example is seen for MS and RA, whose SARS-CoV-2 interacting proteins derived from the same genes (specifically, 49 of the 61 GWAS-associated identical genes). These data suggested the presence of a “core” immune mechanism driving COVID-19 physiopathology shared with most of autoimmune conditions. Conversely, COVID-19 meta-interactome elements belonging to comorbidities’ subsets resulted more specific (T2D 52/66, CAD 12/20, asthma 29/61 unshared genes) ( Figure 5A,B,C). This suggested the possibility that the pathobiological mechanisms underlying the worse prognosis in COVID-19 patients affected by these comorbidities are at least partly independent from the imbalanced immune response to SARS-CoV-2 infection.

**Figure 6.**
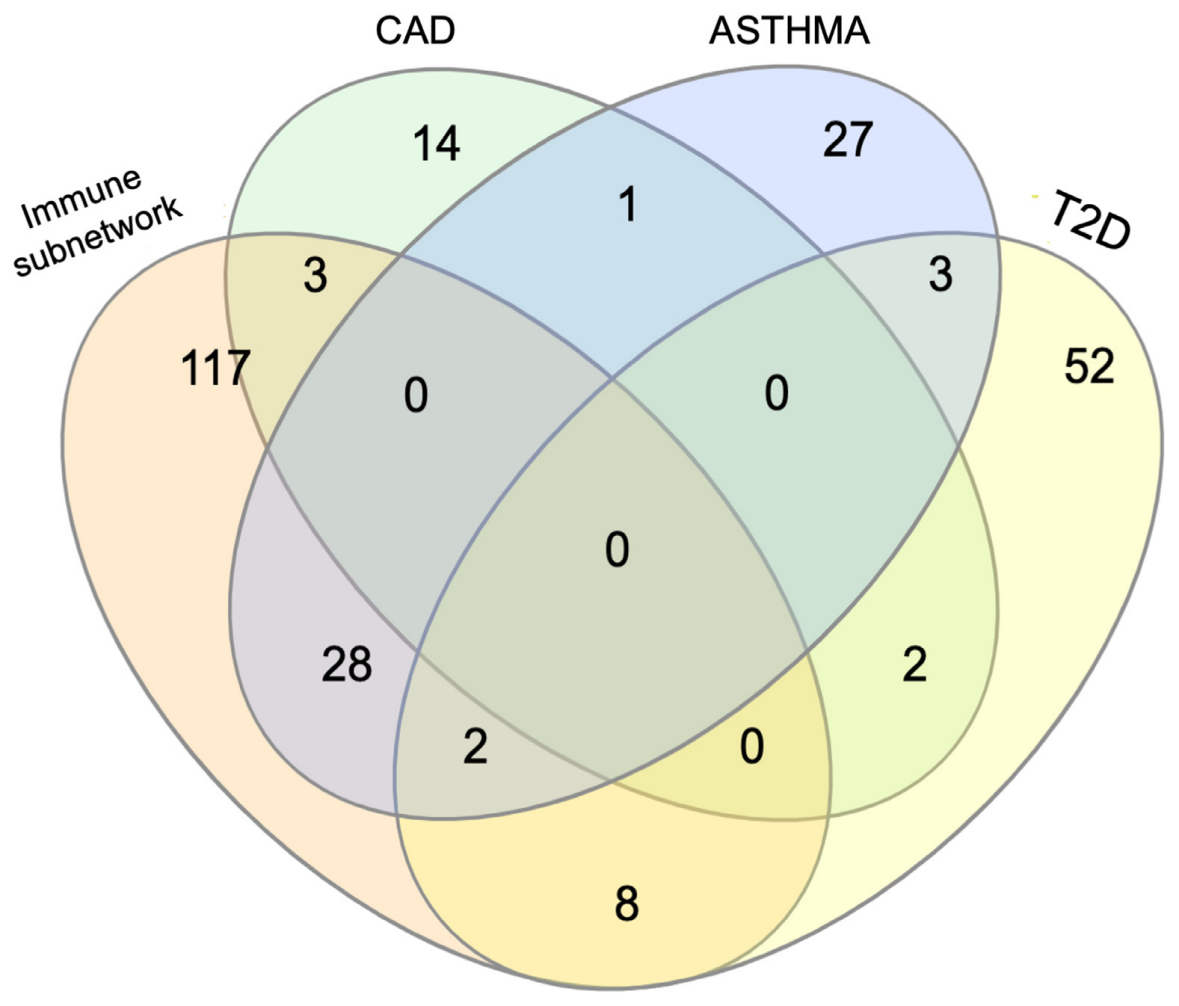
Venn diagram of the overlap between the COVID-19 immune subnetwork and comorbidities’ subnetworks, with the respective number of genes. T2D: Type 2 Diabetes; CAD: Coronary Artery Disease.

**Figure 7.**
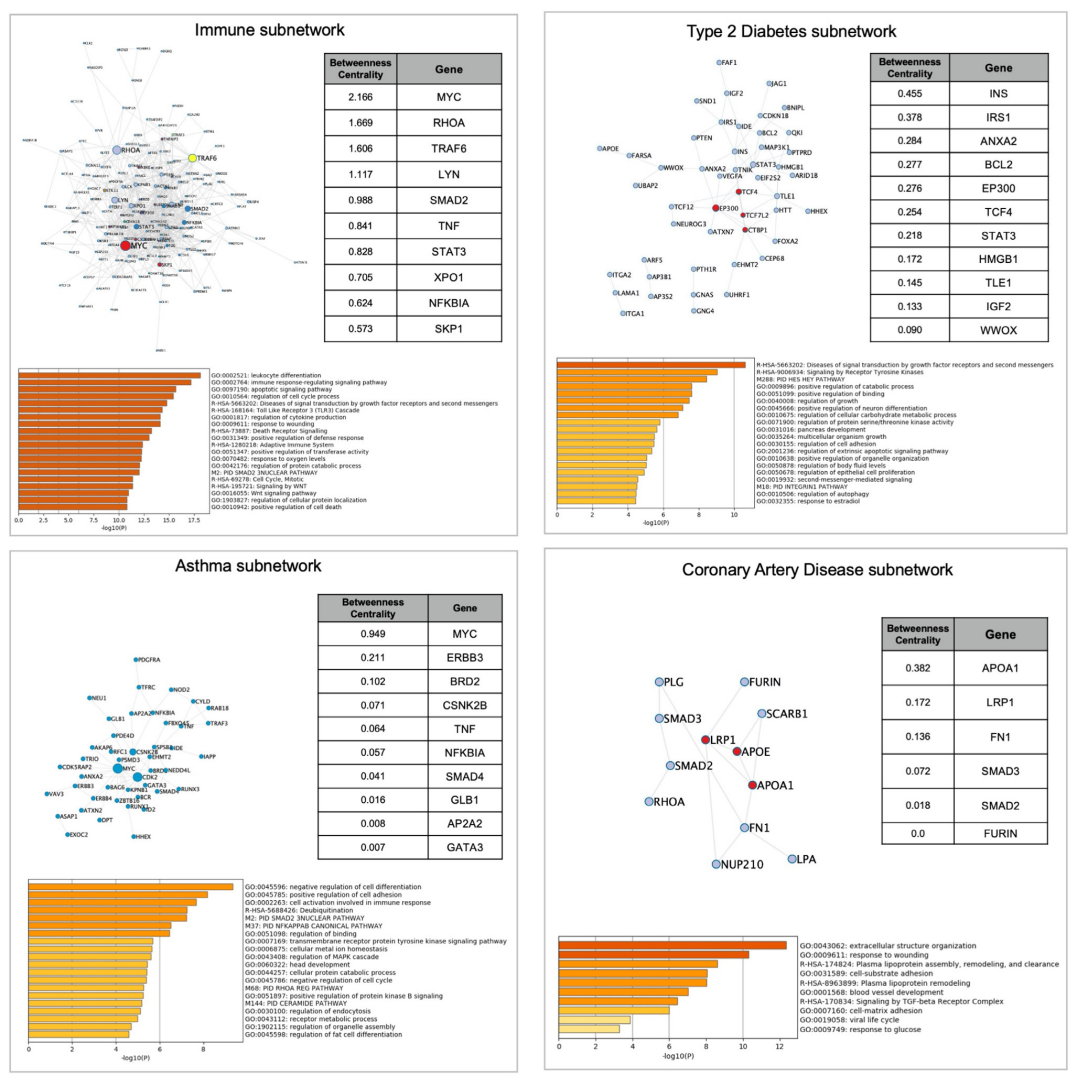
COVID-19 Immune subnetwork an COVID-19 associated comorbidities subnetworks. Each square contains the network representation, the top nodes ranked by Betweenness Centrality (if >0,00) and the pathways with the highest enrichment. Node size is proportional to betweenness centrality; node color represents densely interconnected subsets.

To assess this hypothesis, we next performed a separate analysis of the immune subnetwork, containing genes derived from immunological diseases’ subsets (n=150), and the networks of each comorbidity (Asthma, n=61; T2D, n=66, CA D, n=20). The immune subnetwork pathway analysis confirmed a global enrichment of immunological functions. Betweenness Centrality ranking highlighted again *MYC* as the most relevant node, followed by *RHOA*, encoding for a small GTPase involved in cytoskeleton assembly and nuclear mechanotransduction, and *TRAF6*, a multiacting member of the toll-like receptor (TLR) family, capable of activating MAPK, PI3K, and interferon regulatory factor (IRF) pathways. These nodes also showed a high Betweenness Centrality score in the whole COVID-19 meta-interactome, thus highlighting the importance of immune processes in COVID-19 pathobiology. CAD and T2D only shared a few genes with the immune subnetwork, while, as expected, the overlap with asthma was more extended ( Figure 6). Indeed, pathway and process enrichment analysis of asthma network showed the presence of immunological functions in the top clusters, in contrast to CAD (“ *extracellular structure organization”,”response to wounding”, “Plasma lipoprotein assembly, remodeling, and clearance”)* and T2D (“ *Diseases of signal transduction by growth factor receptors and second messengers”, “Signaling by Receptor Tyrosine Kinases”, “PID HES HEY PATHWAY*”) ( Figure 7). In conclusion, comorbidities subnetworks are largely independent from the immune subnetwork, suggesting that distinct biological processes may interactively contribute to worsening COVID-19 clinical outcomes in these traits.

### COVID-19 meta-interactome gene expression analysis

**Figure 8.**
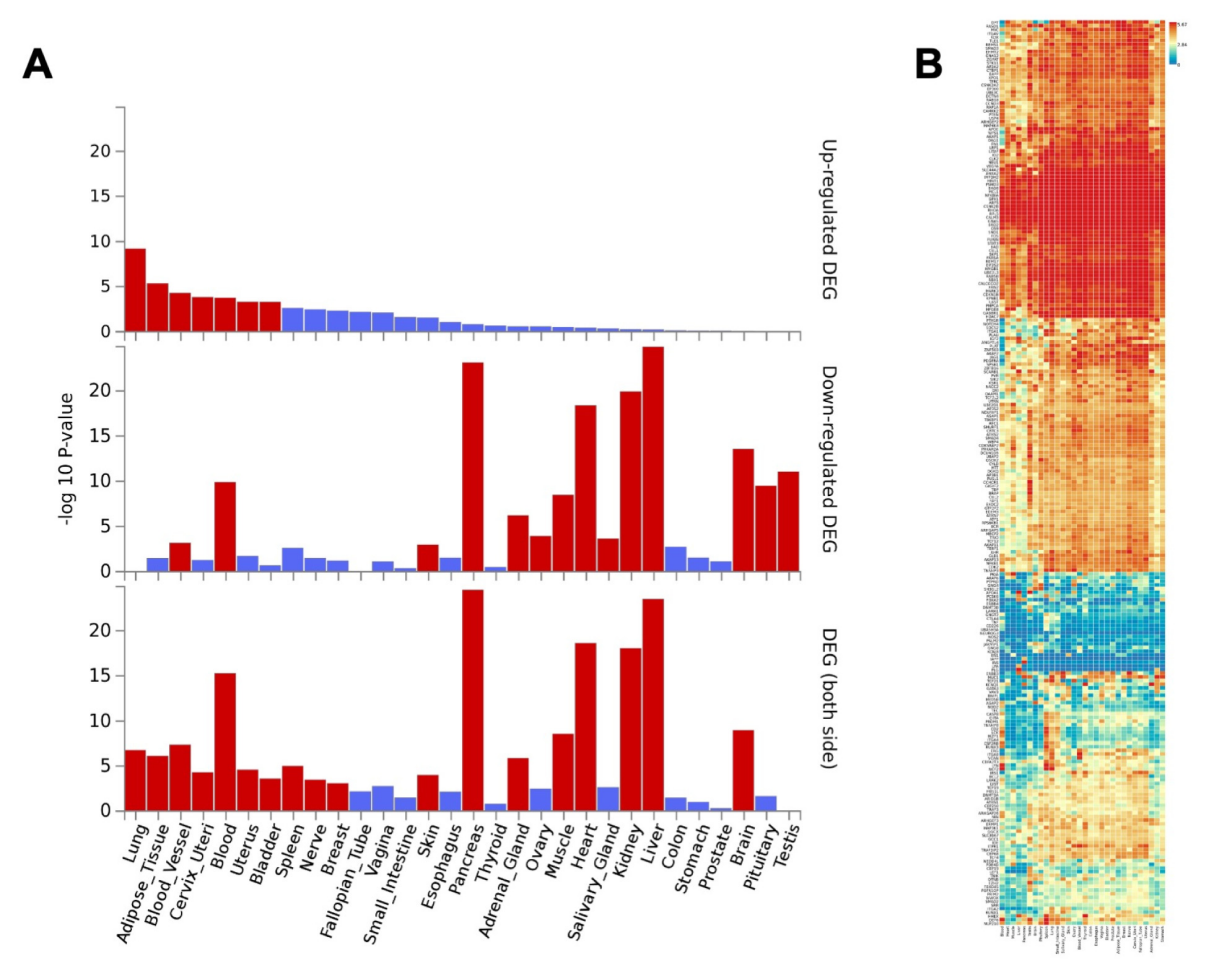
COVID-19 meta-interactome gene expression across human tissues. **A**) Differentially Expressed Genes (DEGs) in GTEx v8 30 tissue types. Significantly enriched DEG sets (Pcorr < 0.05) are highlighted in red: the first graph shows DEGs overexpression, the second DEGs underespression, the third sums both side of regulation. **B**) Gene Expression Heatmap across GTEx v8 30 tissue types. Genes and tissues are ordered by clusters.

To assess the tissue specificity of COVID-19 meta-interactome genes, we conducted a tissue expression pattern analysis based on GTEx v8 RNA-seq data exploiting the FUMA web tool. Results showed a significantly higher expression of COVID-19 meta-interactome genes in lungs, adipose tissue, blood vessels, blood, cervix and uterus. In contrast, the lowest expression was seen in liver, pancreas, kidney and heart ( Figure 8A). Furthering the analysis at the gene level, we found a more prominent tissue specificity of subsets of genes: arteries showed a marked expression of *ITGA8*, *ERG*, *IRS1*; *AGAP2*, *PKIA*, *GNG4*, *KCNJ3* resulted overexpressed in the brain, in contrast to the marked underexpression of *MYC*, *AHR*, *CDK2*, *PLAU*, *TNFAIP3* in this tissue. Results also highlighted a core subset of genes with a marked expression throughout the whole body, as *NFKBIA*, *STAT3* and *FURIN*, a protease known to contribute to SARS-CoV-2 S protein cleavage ^30^ ( Figure 8B). Overall, these expression patterns appeared to relate with the multifaceted clinical manifestation of the disease.

**Figure 9.**
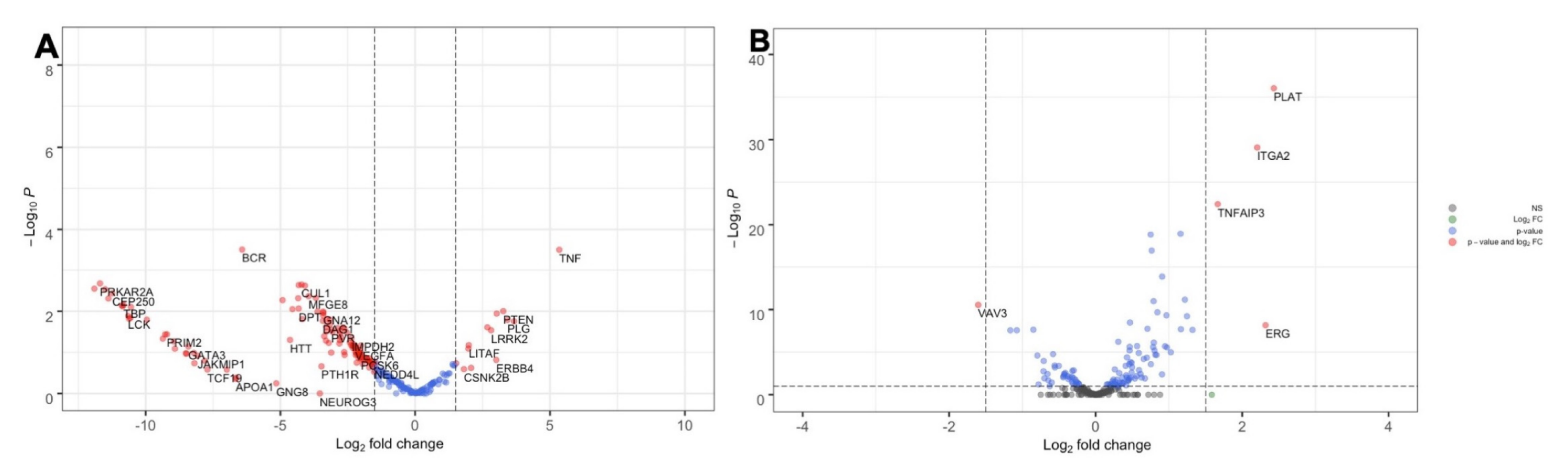
COVID-19 meta-interactome gene expression in SARS-CoV-2 infected human lungs ( **A**) and normal human bronchial epithelial cells treated with IFN-Β ( **B**). Volcano plots displaying the gene expression patterns. The y axis shows adjusted p.value; the x axis shows the Fold Change.

We then investigated if the expression pattern of COVID-19 meta-interactome genes was affected by SARS-CoV-2 infection in humans. To this aim, we reanalyzed the publicly available GSE147507 dataset, containing the transcriptional profiling of lung biopsies of a COVID-19 patient. SARS-CoV-2 infection caused an overall downregulation of COVID-19 meta-interactome genes, with the exception of a small group of genes including TNF ( Figure 9A).

Recent studies suggested that SARS-CoV-2 impairs type I Interferon production, hampering the host’s antiviral response while triggering a sustained chemokine storm ^10, 25, 31, 32^. To explore if the administration of IFN-β could revert the transcriptional profile of COVID-19 meta-interactome genes, we extracted from the GSE14750 the transcriptional profiling results of normal human bronchial epithelial cells treated with human recombinant IFN-β. Indeed, the COVID-19 meta-interactome genes showed a slight upregulation trend, in contrast to the effect of SARS-CoV-2, ( Figure 9B). We then investigated the component of COVID-19 meta-interactome in the Interferome database, an online collection of experimental data regarding Interferon Regulated Genes (IRG): we retrieved experimental evidence that all the 248 COVID-19 meta-interactome genes are regulated by type I interferon.

### Drug target screening

Finally, we investigated whether COVID-19 meta-interactome nodes were suitable targets for drug repositioning. With this aim, we interrogated the Scalable Precision Medicine Oriented Knowledge Engine (SPOKE) ^28^, a continuously updated collection of biomedical data acquired from 25 databases, including CHEMBL, DrugBank and LINCS. We focused our search on the meta-hubs, i.e. the nodes with the highest Betweenness Centrality score in the whole meta-interactome and in the above analyzed subnetworks ( Figure 2 and Figure 7), as their effects extend widely to the network, making them good candidates for therapeutic purposes. We only considered compounds in advanced stage of development (phase ≥3) and approved drugs, aiming at clinical actionability.

At first, we looked for drugs interacting directly with the protein derived from each gene and retrieved 10 proteins targeted by 66 different drugs ( *Extended data* ^13^) Among these appeared the JAK inhibitors Tofacitinib, approved to treat Rheumatoid Arthritis, and Fedratinib, developed for myeloproliferative diseases. This class of compounds is already under clinical investigation for COVID-19, owing to the capacity to interfere with SARS-CoV-2 infection of cells and to reduce cytokine hyperproduction ^33^. Anti-TNF agents, included in our results, follow the same track by blocking a pivotal molecule of the hyperinflammatory response. Among less clinically explored compounds, is Niclosamide that proved to target both LYN and STAT3 in the meta-interactome: it is an approved anti-helminthic drug, which has been recently demonstrated to have very potent antiviral activity against SARS-CoV-2 ^34^.

Considering the effects of SARS-CoV-2 infection on COVID-19 meta-interactome gene expression, we extended the screening in SPOKE to drugs capable of reverting the pattern for each top node. Among these we found anti-diabetic drugs (sitagliptin, pioglitazone, rosiglitazone, canaglifozin), statins (atorvastatin, fluvastatin, simvastatin) and an angiotensin-receptor-blocker (valsartan): these results enhance the pathological relation of COVID-19 with cardiovascular diseases and diabetes, with shared pathways that may benefit from the same interventions.

Results also included antiviral agents, such as ribavirin, which is receiving positive results in COVID-19 management ^35^, and nelfinavir, which has been demonstrated to inhibit SARS-CoV-2 infection and replication both *in silico* and *in vitro* ^36, 37^; along with these, anti-inflammatory, immunomodulatory and immunosuppressive agents also emerged. Of interest is the presence of progesterone, estradiol, selective estrogen receptor modulators (SERMs: Tamoxifen, Bazedoxifene, Raloxifene) and anti-androgen medications (finasteride, flutamide): these results suggest that the effects of diverse steroidal hormones at the transcriptional level may underlie the sex discrepancy seen in COVID-19 severity.

In conclusion, we found multiple repurposable molecules targeting SARS-CoV-2-host interactions: to organize the results, we implemented a “pleiotropy ranking” of the whole list of compounds, on the basis of the multiplicity of positive effects each drug is expected to have on COVID-19 meta-interactome both at the protein and at the gene expression level ( *Extended data* ^13^).

## Discussion

We found a significant enrichment of SARS-CoV-2 interactors among proteins coded by genes associated with autoimmune diseases. Notably, similar enrichments were not found when we considered other HCoVs or other respiratory human viruses as controls. This finding suggests that SARS-CoV-2 is able to activate an auto-aggressive response in a subset of infected people and that such activation may be influenced by a genetic, virus-specific, predisposition of the host. The result has implications for risk-assessment and, possibly, for future vaccination policies.

We also found an enrichment of SARS-CoV-2 interactors among proteins coded by genes associated with comorbidities - T2D, asthma and CAD - that contribute to the severity of the disease. Interestingly, T2D and asthma have an inflammatory pathophysiology that is absent or less pronounced in those comorbidities that did not show clear enrichments. Also, in the case of T2D, asthma and CAD, the results seem to be specific for SARS-CoV-2 compared to other viruses.

Compared to SARS-CoV-2, in the other highly pathogenetic H-CoVs the link with autoimmunity was definitely looser, pointing out to pathobiological differences between infections that share clinical similarities. The higher lethality rate of MERS-CoV (~30%) and SARS-CoV (~10%), the broader cytopathic effects across tissues, and the clearer tropism towards immune cells (macrophages, T lymphocytes, dendritic cells) configure them as ancestors struggling to adapt to the human host after the zoonotic transmission ^38– 40^. This is in line with the higher infectious rate of SARS-CoV-2, as well as with its divergent cytokine profile compared to other HCoVs ^41^. Indeed, the exuberant immune response underlying the severe pneumonia and the multiorgan failure distinguishes COVID-19 from the other respiratory diseases caused by viruses ^42^. 

When we considered the tissue expression of the COVID-19 meta-interactome in the GTEx resource, lung, blood and blood vessels emerged, in agreement with the main targets of the infection. Concerning the lung, the ‘ *in silico*’ prediction found a significant correspondence with data *‘in vivo’* coming from a recent study on the transcriptome from infected patients ^25^: most of the components of COVID-19 meta-interactome were down-regulated. This gene expression profile was consistent with an inappropriate inflammatory response (low levels of type I and III interferons, elevated chemokines and high expression of IL-6), suggesting reduced innate antiviral defenses coupled with exuberant inflammatory cytokine production. This pattern suggests that the expression of most components of COVID-19 meta-interactome in the lung is negatively affected by the infection and positively regulated by exogeneous type I interferon: this was confirmed by our reanalysis of transcriptional profiles of normal human bronchial epithelial cells treated with human recombinant interferon-β ^25^, and by the presence of COVID-19 meta-interactors in the Interferome, the data set of interferon-regulated genes ^27^. Moreover, the encouraging results of a recent trial with interferon β-1b in patients with COVID-19 support this view ^35^.

It is intriguing to note that interferon β is a pivotal first-line therapy for MS ^43^, the autoimmune disease with the strongest enrichment in SARS-CoV-2 interactors. It will be interesting to verify the clinical course of MS patients infected by SARS-CoV-2, receiving IFN-β or other immunotherapies ^44^.

The construction of a COVID-19 meta-interactome allowed us to identify densely interconnected clusters of interactors encompassing gene products of autoimmune diseases and comorbidities, and highly connected nodes, exerting a critical control on the network. These meta-hubs, mainly linked to immunological pathways (immune response regulation, cytokine production, interleukin signaling, neutrophils pathway) may represent clinically actionable interactors, suitable for anti-virus drug discovery or repurposing. Among the main pathways resulting from the analysis of the meta-interactome, we found an enrichment for hemostasis pathways. This result supports the dysfunction of coagulation that is frequently observed in complicated cases of SARS-CoV-2 infection. Our recent analysis on genome-wide MS association data and coagulation showed an over-connectivity between the two networks ^45^, suggesting that the link between hyper-inflammatory state and activation of coagulative pathway is shared by COVID-19 and MS.

The presence of more independent sub-networks of T2D and CAD within the meta-interactome suggests the existence of two mechanisms underlying COVID-19 course: one is autoinflammatory, SARS-CoV-2-specific, and under the influence of the genetic background of the host; the other is influenced by the presence of other diseases that synergize with the virus in a non-specific way as they do with many other microbial and non-microbial diseases.

The drug repurposing screening we performed to target COVID-19 meta-interactome key nodes suggest the possibility to tailor the therapeutic framework including drugs targeting the immune subnetwork together with compounds interfering with the specific comorbidity subnetwork.

Our analysis presents some limitations. First, the lack of a SARS-CoV-2 interactome integrating predicted and confirmed protein-protein interactions (PPIs) – as for the other viruses we considered – caused us to use a different method to expand the *in vitro* demonstrated network. To homogenize this mismatch, we included in the analysis multiple levels of high-confidence, only physical PPIs inferred by an optimally referenced bioinformatic resource. Additionally, we built our analysis at the gene level, and the single-nucleotide variants specifically associated with each of the considered conditions may affect the resultant virus-host interaction, either in a positive or a detrimental fashion.

Genome-wide association studies, as well as genetic profiling of rare naturally-resistant individuals and “unexpectedly” severe cases, are needed to clarify the possible role played by the host genetic variability in the clinical outcome of SARS-CoV-2 infection ^46, 47^. In this context, recent results highlighted 2 loci associated to respiratory failure in COVID-19 ^48^, that were not included in our analysis. However, the proteins coded by genes associated to these loci, showed direct interaction with some members of our meta-network: FYCO1 with XPO1, EP300, CYLD; LZTFL1 with CEP250 ^49– 52^. Indeed, our study complements these approaches, proxying genetics to clarify COVID-19 pathophysiology, and provides information that may increase the probability of success in the development of new treatments.

## Data availability

### Underlying data

All data underlying the results are available as part of the article and no additional source data are required.

### Extended data

Open Science Framework: SARS-CoV-2 meta-interactome suggests disease-specific, autoimmune pathophysiologies and therapeutic targets: extended data. https://doi.org/10.17605/OSF.IO/82T5Q ^13^

- SARS-COV-2 meta-interactome genes list.csv (List of interacting genes)
- SARS-COV-2 meta interactome extended data.xlsx (Viral interactomes size and sources, gene sets compositions, gene sets/interactomes intersections numbers, meta-interactome composition, drug target screening and drug pleiotropy ranking)
- Viral interactomes composition..csv (Composition of viral interactomes)

Data are available under the terms of the Creative Commons Zero "No rights reserved" data waiver (CC0 1.0 Public domain dedication).
