# Supplementary material for: SARS-CoV-2 meta-interactome suggests disease-specific, autoimmune pathophysiologies and therapeutic targets
Source: F1000Res. 2020 Aug 17;9:992. [Version 1] doi: 10.12688/f1000research.25593.1 (PMC7791351; doi:10.12688/f1000research.25593.1)
Supplement: Peer review history

# Peer review: Reviewer response for version 1

**DOI:** 10.5256/f1000research.28245.r76689

**Published:** 2021-01-07

**Authors:** Yongming Sang

**Affiliations:** 1 Department of Agricultural and Environmental Sciences, College of Agriculture, Tennessee State University, Nashville, TN, USA

The manuscript describes a computational study of SARS-CoV2 interactome and its association with comorbid situations, especially several autoimmune diseases. The findings from different datasets, converge a causal relationship of SARS-CoV2 infection, particularly during severe COVID-19 progression, with an autoimmune regulation as shown in several autoimmune diseases. Although this kind of autoimmune situation has been associated with COVID-19 progression in previous studies, a meta-interactome study as presented strengths the observation and is informative for targeting prophylactic design and drug repurposing as indicated. I pretty much agree with the second reviewer regarding the pitfalls of presentation and improving readability. Additional concerns include:

1. The interactome varies during the different stages of COVID-19 progression, the autoimmune manifestation is mostly associated with severe COVID-19 patients. The enrichment of interactome loci as shown in Figure 1 is need to correlate to the status of COVID-19 severity (Such as classified in a Yale IMPACT study Lucas C., *et al.* Nature 584, 463–469 (2020)).
2. The description of SARS-CoV2’s suppression of interferon response does not well reflect the full scenario of dysregulated interferon response during the viral infection and COVID-19 progress. Several studies actually determined robust interferon response COVID-19 patients. This also reflected by all the 248 meta-interactome genes are IRGs.  Please refer to some further references to revise the relevant description:
  Lopez L, Sang PC, Tian Y, Sang Y. Dysregulated Interferon Response Underlying Severe COVID-19. Viruses. 2020 Dec 13;12(12):1433. ^1^
  Lee, J.S.; Park, S.; Jeong, H.W.; Ahn, J.Y.; Choi, S.J.; Lee, H.; Choi, B.; Nam, S.K.; Sa, M.; Kwon, J.S.; *et al.* Immunophenotyping of COVID-19 and influenza highlights the role of type I interferons in development of severe COVID-19. *Sci. Immunol.* **2020**, *5*, eabd1554. ^2^
  Lucas, C.; Wong, P.; Klein, J.; Castro, T.B.R.; Silva, J.; Sundaram, M.; Ellingson, M.K.; Mao, T.; Oh, J.E.; Israelow, B.; *et al.* Longitudinal analyses reveal immunological misfiring in severe COVID-19. *Nature* **2020**. ^3^
3. Other recent GWAS studies have not been included for analysis and discussion. In addition to gene loci in IFN-signaling, multiple gene loci in chemokine signaling were reproducibly associated with COVID-19.  The following key references need to be considered for interpreting/discussing their *in silico* findings:
  McCoy, K.; Peterson, A.; Tian, Y.; Sang, Y. Immunogenetic Association Underlying Severe COVID-19. Vaccines 2020, 8, 700. ^4^
  Severe Covid-19 GWAS Group; Ellinghaus, D.; Degenhardt, F.; Bujanda, L.; Buti, M.; Albillos, A.; Invernizzi, P.; Fernández, J.; Prati, D.; Baselli, G.; *et al.* Genomewide Association Study of Severe Covid-19 with Respiratory Failure. N. Engl. J. Med. 2020, 383, 1522–1534. ^5^
  Pairo-Castineira, E.; Clohisey, S.; Klaric, L.; Bretherick, A.; Rawlik, K.; Parkinson, N.; Pasko, D.; Walker, S.; Richmond, A.; Fourman, M.H.; *et al. *Genetic mechanisms of critical illness in Covid-19. medRxiv 2020. ^6^
  Zeberg, H.; Pääbo, S. The major genetic risk factor for severe COVID-19 is inherited from Neanderthals. Nature 2020. ^7^

**Minor issues: **

1. ‘drug repurposing’ is a more meaningful keyword; “impairs (or low levels of) type I Interferon production” are not accurate. Please see Point 2 above. Please define and make consistent the abbreviations used in the text. Such as “H-CoV/HCoV”.
2. Figure 8A, what is the legend for either blue bars or red bars. Seems the same thing, and does DEG for both sides mean total DEG?
3. As indicated all 248 interactome gene loci are IRGs, i.e. IFN signaling is the core of the profiled interactome, why the drug repurposing study identifies no IFN-based prophylactics?    

Is the work clearly and accurately presented and does it cite the current literature?

Partly

If applicable, is the statistical analysis and its interpretation appropriate?

Yes

Are all the source data underlying the results available to ensure full reproducibility?

Partly

Is the study design appropriate and is the work technically sound?

Yes

Are the conclusions drawn adequately supported by the results?

Partly

Are sufficient details of methods and analysis provided to allow replication by others?

Partly

Reviewer Expertise:

Immunogenetics, antiviral immunology, interferon biology, immunometabolism

I confirm that I have read this submission and believe that I have an appropriate level of expertise to confirm that it is of an acceptable scientific standard, however I have significant reservations, as outlined above.

## References

[1] : Dysregulated Interferon Response Underlying Severe COVID-19. *Viruses*.2020;12(12) : 10.3390/v12121433 10.3390/v12121433 PMC776412233322160

[2] : Immunophenotyping of COVID-19 and influenza highlights the role of type I interferons in development of severe COVID-19. *Science Immunology*.2020;5(49) : 10.1126/sciimmunol.abd1554 10.1126/sciimmunol.abd1554 PMC740263532651212

[3] : Longitudinal analyses reveal immunological misfiring in severe COVID-19. *Nature*.584(7821) : 10.1038/s41586-020-2588-y 463-469 10.1038/s41586-020-2588-y 32717743PMC7477538

[4] : Immunogenetic Association Underlying Severe COVID-19. *Vaccines (Basel)*.2020;8(4) : 10.3390/vaccines8040700 10.3390/vaccines8040700 33233531PMC7711778

[5] : Genomewide Association Study of Severe Covid-19 with Respiratory Failure. *New England Journal of Medicine*.2020;383(16) : 10.1056/NEJMoa2020283 1522-1534 10.1056/NEJMoa2020283 PMC731589032558485

[6] : Genetic mechanisms of critical illness in Covid-19. *Nature*.2020; 10.1038/s41586-020-03065-y 10.1038/s41586-020-03065-y 33307546

[7] : The major genetic risk factor for severe COVID-19 is inherited from Neanderthals. *Nature*.587(7835) : 10.1038/s41586-020-2818-3 610-612 10.1038/s41586-020-2818-3 32998156

# Peer review: Reviewer response for version 1

**DOI:** 10.5256/f1000research.28245.r72616

**Published:** 2020-10-19

**Authors:** Mohd M. Khan

**Affiliations:** 1 Booz Allen Hamilton Inc., McLean, VA, USA

In this study, the authors conducted (meta) interactome analyses to deduce the molecular interactions between the host and virus that shape the severe acute respiratory syndrome coronavirus 2 (SARS-CoV-2) infection in humans. Such analyses were further leveraged to uncover the molecular pathways and associated genes that may play roles in co-morbidities (e.g., type 2 diabetes (T2D), coronary artery diseases (CAD), immune-related conditions, and respiratory diseases such as asthma) that may impact disease severity and overall outcomes. The authors concluded that a significant enrichment of SARS-CoV-2 interactome was tied to the genes that have established roles in multiple autoimmune diseases, and share significant interactions with CAD, T2D, and asthma co-morbidities. Furthermore, they conducted *in silico *drug target screening and proposed several molecules that may target SARS-CoV-2-host interactions and inflammatory pathways associated with infection. To validate some of the meta-interactome genes identified in their analysis, authors leveraged the RNAseq data ((GEO: GSE147507; data is from a peer-reviewed study that proposed that the reduced innate antiviral defenses (low levels of type I and III interferons (IFNs)) and inflammatory cytokine production (elevated chemokines and high expression of IL-6) may drive COVID-19 infection) and found reduced expression of meta-interactome genes in human lungs post-SARS-Cov2-infection and proposed roles of Type I IFNs.

 Overall, this exploratory study is interesting as reported results are in conformity with the earlier published work on coronaviruses and their associated pathophysiologies; introduction, methodology, results, and discussion sections are well-described and appropriately cited. Given there is additional information available, including on the potential of IFN therapies (stand-alone and/or in a combination with other drugs; eg., ACTT-3 trial), authors may cite those and provide additional context for the exploratory results provided here.

Is the work clearly and accurately presented and does it cite the current literature?

Yes

If applicable, is the statistical analysis and its interpretation appropriate?

Yes

Are all the source data underlying the results available to ensure full reproducibility?

Yes

Is the study design appropriate and is the work technically sound?

Yes

Are the conclusions drawn adequately supported by the results?

Yes

Are sufficient details of methods and analysis provided to allow replication by others?

Yes

Reviewer Expertise:

Systems Proteomics; Host-pathogen Biology of Infectious Diseases

I confirm that I have read this submission and believe that I have an appropriate level of expertise to confirm that it is of an acceptable scientific standard.

# Peer review: Reviewer response for version 1

**DOI:** 10.5256/f1000research.28245.r69604

**Published:** 2020-09-14

**Authors:** Siddhartha Mahanty

**Affiliations:** 1 The Peter Doherty Institute for Infection and Immunity, The Royal Melbourne Hospital, University of Melbourne, Melbourne, Vic, Australia

The authors have conducted a comprehensive interactome analysis of SARS-CoV-2 infections in humans using a number of conventional and recently developed computational approaches. Genes and pathways of significance identified by this interactome analysis were matched with genes associated with comorbid disease (type-2 diabetes, cardiovascular disease, chronic obstructive pulmonary disease etc) using a similar approach to identify a meta-interactome between COVID-19 disease and the comorbidities associated with disease severity. They demonstrate that the SARS-CoV-2-host interactors concentrated in immunological pathways and were strongly associated with autoimmunity and three comorbidities of prognostic significance, diabetes, coronary artery disease, and asthma. Analysis of RNAseq data from post-mortem lung tissue in a single patient demonstrated lower expression of meta-interactome genes identified in their network analysis. Finally, the authors speculate on potential drug targets, among currently used drugs in the autoimmunity and diabetes domains that may be of interest for the management of SARS-CoV-2 infections.

 The rationale for the computational approach, selection of analysis software, and methodology used in the network analysis are described adequately, although I would recommend clearer explanations with some background information about each approach for the general readership unfamiliar with this field. The results are consistent with the proposed pathophysiology of COVID-19 disease, and there are no real surprises in this exploratory research. This raises a question as to the importance of the sample size from which they have available data. How much could a larger sample size, particularly samples from infected individuals with a broader range of clinical presentations influence their results? Many conclusions appear to be speculative or based on small (in some cases, single) studies and patient numbers – bias is frequently unavoidable in such small, select datasets. These will presumably require validation with larger data sets. Nevertheless, the comprehensive meta-interactome undoubtedly serves as a good framework for a generation of hypotheses and starting points for research on pathogenesis and treatment of COVID-19 disease.

 General comments on presentation and content:

1. Much of the data presented in figures are direct outputs of computational analysis. The manuscript would benefit from more creative displays for the general readership, such as a schematic encompassing the study design and methodology and the type of data generated with each procedure.
2. In describing the analysis of interactomes between SARS-CoV-2 and disease-associated genes (end of Page 3) the term “overlapped” is unclear. Do they mean genes that are regulated similarly in the two conditions? A clearer explanation of the procedure is needed for readers unfamiliar with interactome methodology. 
3. While mention is made of the 6 other coronaviruses and 4 other respiratory viruses as reference organisms, how they were used in the analysis is unclear. 
4. Suggestions to the research community about the type of data that are required to fill knowledge gaps or refine the interactome would be helpful in directing ongoing research efforts.

Is the work clearly and accurately presented and does it cite the current literature?

Yes

If applicable, is the statistical analysis and its interpretation appropriate?

I cannot comment. A qualified statistician is required.

Are all the source data underlying the results available to ensure full reproducibility?

Yes

Is the study design appropriate and is the work technically sound?

Yes

Are the conclusions drawn adequately supported by the results?

Partly

Are sufficient details of methods and analysis provided to allow replication by others?

Yes

Reviewer Expertise:

Parasite immunology, Immuneregulation, viral hemorrhagic fevers, antibody functions

I confirm that I have read this submission and believe that I have an appropriate level of expertise to confirm that it is of an acceptable scientific standard, however I have significant reservations, as outlined above.
